# TIMP1 and MMP9 are predictors of mortality in septic patients in the emergency department and intensive care unit unlike MMP9/TIMP1 ratio: Multivariate model

**DOI:** 10.1371/journal.pone.0171191

**Published:** 2017-02-13

**Authors:** Maria Eugenia Niño, Sergio Eduardo Serrano, Daniela Camila Niño, Diana Margarita McCosham, Maria Eugenia Cardenas, Vivian Poleth Villareal, Marcos Lopez, Antonio Pazin-Filho, Fabian Alberto Jaimes, Fernando Cunha, Richard Schulz, Diego Torres-Dueñas

**Affiliations:** 1 Department of Public Health, Medicine Program, Faculty of Health Sciences, Universidad Autónoma de Bucaramanga, Bucaramanga, Santander, Colombia; 2 Department of Pharmacology, Medicine Program, Faculty of Health Sciences, Universidad Autónoma de Bucaramanga, Bucaramanga, Santander, Colombia; 3 Microbiology and Inmunology Department, Medicine Program, Faculty of Health Sciences, Universidad Autónoma de Bucaramanga, Bucaramanga, Santander, Colombia; 4 Biotechnology Department, Enterprise Technology Center, Fundación Cardiovascular de Colombia, Bucaramanga, Santander, Colombia; 5 Department of Medical Clinics, Emergency unit, Faculty of Medicine, Universidade de Sao Paulo, Ribeirao preto, Sao Paulo, Brazil; 6 Department of Internal Medicine, School Of Medicine, Universidad de Antioquia, Medellin, Antioquia, Colombia; 7 Department of Pharmacology, Faculty of Medicine, Universidade de Sao Paulo, Ribeirao preto, Sao Paulo, Brazil; 8 Departments of Pediatrics and Pharmacology, Faculty of Medicine & Dentistry, University of Alberta, Edmonton, Alberta, Canada; University of South Alabama Mitchell Cancer Institute, UNITED STATES

## Abstract

**Introduction:**

Matrix metalloproteinases and tissue inhibitors of metalloproteinases could be promising biomarkers for establishing prognosis during the development of sepsis. It is necessary to clarify the relationship between matrix metalloproteinases and their tissue inhibitors. We conducted a cohort study with 563 septic patients, in order to elucidate the biological role and significance of these inflammatory biomarkers and their relationship to the severity and mortality of patients with sepsis.

**Materials and methods:**

A multicentric prospective cohort was performed. The sample was composed of patients who had sepsis as defined by the International Conference 2001. Serum procalcitonin, creatinine, urea nitrogen, C-Reactive protein, TIMP1, TIMP2, MMP2 and MMP9 were quantified; each patient was followed until death or up to 30 days. A descriptive analysis was performed by calculating the mean and the 95% confidence interval for continuous variables and proportions for categorical variables. A multivariate logistic regression model was constructed by the method of intentional selection of covariates with mortality at 30 days as dependent variable and all the other variables as predictors.

**Results:**

Of the 563 patients, 68 patients (12.1%) died within the first 30 days of hospitalization in the ICU. The mean values for TIMP1, TIMP2 and MMP2 were lower in survivors, MMP9 was higher in survivors. Multivariate logistic regression showed that age, SOFA and Charlson scores, along with TIMP1 concentration, were statistically associated with mortality at 30 days of septic patients; serum MMP9 was not statistically associated with mortality of patients, but was a confounder of the TIMP1 variable.

**Conclusion:**

It could be argued that plasma levels of TIMP1 should be considered as a promising prognostic biomarker in the setting of sepsis. Additionally, this study, like other studies with large numbers of septic patients does not support the predictive value of TIMP1 / MMP9.

## Introduction

Sepsis is a complex, multisystemic, and variable clinical process, produced by pathogenic microorganisms causing a deleterious systemic response in the host [1]. In the United States, the incidence of severe sepsis is estimated to be 300 cases per 100 000 persons [2]. Many resources and research projects have focused on the study of biomarkers for sepsis that would allow early diagnosis of this syndrome, improve its course, and decrease morbidity and mortality. In this regard, there have been multiple biomarkers used in the diagnosis and stratification of sepsis, including interleukins, cytokines, C-reactive protein, procalcitonin, lipopolysaccharide binding protein, coagulation factors, atrial natriuretic peptide and brain natriuretic peptide (ANP and BNP respectively), among many others [3,4].

Matrix metalloproteinases (MMPs) are proteinases that participate in Extracellular Matrix (ECM) degradation; the activities of MMPs are accurately regulated at the level of transcription and activation of precursor zymogens [5]. Tissue inhibitors of metalloproteinases (TIMPs) are specific inhibitors of MMPs that participate in controlling local activities in tissues [6]. It has been described that the balance of the extracellular matrix depends largely on the close interaction between MMPs and TIMPs. Furthermore, it has also been reported that the ECM plays a very important role in different cells and tissues [7]. In addition, they are involved in different pathophysiological processes such as coronary syndrome [8], vascular disease [9], heart failure [10] and immunopathogenesis [11], among others.

Matrix metalloproteinases (MMPs) and tissue inhibitors of metalloproteinases (TIMPs) could be promising biomarkers for establishing prognosis during the development of sepsis [12]. Specifically, Hoffman et al., observed an association between mortality and elevated plasma levels of MMP9, TIMP2, TIMP1 in septic patients. They also found significantly elevated levels of TIMP1 in non-survivors compared with survivors. However, they did not find the same relationship with MMP9 levels among these groups [13]. In addition, they established a predictive cut-off TIMP1 serum value of 3200 ng/mL, which was associated with a 4.5 times higher risk of mortality [13]. Unlike Lorente et al., found low levels of MMP9 and a lower MMP9/TIMP1 ratio in non-surviving septic patients [14]. Interestingly, they also found a cutoff plasma concentration of TIMP1 at 531 ng/mL [14], which was considerably lower than that described by Hoffmann et al., 2006 [13]. Recently, in a study Involving 192 Patients with severe sepsis it was observed association between TIMP1 / MMP9 ratio and mortality in a predictive model at 30 days follow-up [15]. However, Wang et al., 2014, in a study of 360 patients (180 sepsis, 90 severe sepsis and 90 septic shock), did not find a predictive value for MMP9/TIMP1 ratio. They only found a predictive value for mortality and AKI with significantly elevated levels of TIMP1 [16].

Considering this, it is necessary to clarify the relationship between matrix metalloproteinases and their tissue inhibitors (TIMPs). Importantly, given the complex pathophysiology of sepsis, and after many isolated biomarker studies, it is recommended that current studies should take into account a set of biomarkers and their association with severity scales such as APACHE and SOFA and other outcomes [17]. Therefore, we have performed a multivariate analysis, which includes matrix metalloproteinases and their tissue inhibitors (TIMPS), along with other variables: biochemical, clinical, epidemiological and severity scales. We conducted a cohort study with 563 septic patients, in order to elucidate the biological role and significance of these inflammatory biomarkers and their relationship to the severity and mortality of patients with sepsis.

## Materials and methods

### Type of study

An analytical prospective cohort multicentric study was performed, which included for health centers in the city of Bucaramanga (medium size city in Colombia). The ethics committee of the Autonomous University of Bucaramanga (approval number 0056/2009) and each of the four participating institutions approved the study(Investigation Ethics committee of “Empresa Social del Estado Hospital Universitario de Santander”(HUS), Ethics committee of the chicamocha clinic, Ethics committee of Los Comuneros clinic, and the ethics committee of “Fundación Oftalmologica de Santander/Clinica Carlos Ardila Lulle” (FOSCAL)). The population studied was composed of patients over the age of 18 who had sepsis (as defined by the International Sepsis Conference 2001 [1] at the time of study entry). Inclusion criteria: septic patients with the age of 18 or older, which were in the emergency department or intensive care unit. Exclusion criteria: patients under the age of 18 and patients 18 or older with over 72 hours from the initial diagnosis of sepsis.

Before participating in the study, an informed consent was signed by the patient or their legal representative if their mental status did not allow them to personally consent. After obtaining the consent, a blood sample was taken (20 ml) for quantification of serum procalcitonin, creatinine, urea nitrogen, C-reactive protein, TIMP1, TIMP2, MMP2 and MMP9; each patient was followed until death or up to 30 days, with a registration of their vital signs and other parameters for calculating APACHE II, SOFA and Charlson scales.

### Sampling

Blood samples were obtained from five clinics in the city. Venous blood samples were collected in serum separator tubes and into tubes containing ethylenediaminetetraacetic acid (EDTA).

Once the samples were obtained, they were left to stand at room temperature for 30 minutes and subsequently centrifuged at 1000g for 15 minutes. Serum samples were removed and stored at ≤ -20° C. The biomarkers analyses were carried out in the facilities of the Specialized Clinical Laboratory of Bucaramanga and the facilities of the Autonomous University of Bucaramanga.

### Quantification of MMP9, MMP2, TIMP1 and TIMP2

The levels of MMP9, MMP2, TIMP1 and TIMP2 were determined with the use of human Quantikine Immunoassay kits (R & D Systems). The procedure was performed according to the manufacturer's instructions. The functional scale range for the MMP9 assay was set from 0.25 to 16 ng / mL, for MMP2 from 0.78 to 50 ng / mL, for TIMP1 from 0.156 to 10 ng / mL, and for TIMP -2 from 0.156 to 10 ng / mL.

### Quantification of N-terminal B-type Natriuretic Peptide (proBNP)

ProBNP concentrations were obtained through a quantitative electrochemiluminescence immunoassay designed for use in the Elecsys and cobas immunoassay analyzers. The procedure was performed according to the manufacturer's instructions. The functional scale range set by the manufacturer of the test was 0.02 to 100 ng / mL and its functional sensitivity was 50 pg / mL.

### Quantification of Procalcitonin (PCT)

ROCHE (electrochemiluminescent assay)—the test "Elecsys BRAHMS PCT, COBAS was used for the quantitative determination of PCT. The procedure was performed according to the manufacturer's instructions. The scale range established by the manufacturer was 0.02 to 100 ng / mL and its functional sensitivity is ≤ 0.06 ng / mL.

Clinical data presented by the test indicate that a value lower than 0.5 ng / mL represents a low risk of severe sepsis and / or septic shock and a value higher than 2.0 ng / mL represents a high risk of severe sepsis and / or septic shock.

### Statistical analysis

A distribution analysis of the continuous variables was conducted using graphical tests (with the command pnorm in stata13) and it was concluded that continuous variables had an approximately normal distribution. the test used to compare survivors and non survivors was a test of mean difference (performed in stata 13 by the command ttest). A descriptive analysis was performed by calculating the mean and the 95% confidence interval for continuous variables and proportions for categorical variables (Performed in stata 13 by the command prtest).

A receiver operating characteristic (ROC) analysis or ROC curve was performed for MMPs and TIMPs as predictors of mortality. Bivariate analysis with univariate logistic regression (mortality as a dependent variable and each of the other variables as independent variables of the study).

A multivariate logistic regression model was constructed by the method of intentional selection of covariates, which is a backwards method described in the book of Hosmer and Lemeshow “Applied Logistic Regression” [18]. initially including variables with P <0.2 (in the final model only those with P<0.05 and confounding variables were included) in the bivariate analysis obtaining a final model with 4 statistically significant variables and a confounding variable, which was evaluated by the Hosmer and Lemeshov test and by ROC analysis of the model to assess its predictive ability.

## Results

Graphic tests of the normality of the variables were performed and it was found that the variables followed an approximately normal distribution; therefore, means with their respective confidence intervals were used.

In Table 1, we can see the description of the population with regards to the variables collected stratified by the survival status at 30 days.

**Table 1:** Description of the patient population.

Variable	PATIENT STATUS AT 30 DAYS						TOTAL			P
	SURVIVOR			NON-SURVIVOR						
	MEAN	CI 95%		MEAN	CI 95%		MEAN	CI 95%		
MALE*	51.7	47.6	55.8	53.3	42.8	63.5	52.0	48.1	55.8	0.7797
AGE	55.9	54.3	57.5	68.5	65.1	71.9	57.6	56.2	59.1	<0.0001
SOFA	1.7	1.5	1.9	4.7	3.8	5.6	2.1	1.9	2.4	<0.0001
APACHE II	9.4	8.9	9.9	16.8	14.9	18.8	10.4	9.9	11.0	<0.0001
CHARLSON	1.6	1.4	1.7	2.7	2.1	3.3	1.7	1.6	1.9	<0.0001
CRP	130.3	121.4	139.2	166.6	135.2	198.0	133.8	125.2	142.4	0.0142
Procalcitonin	6.1	4.6	7.6	11.1	5.6	16.7	6.7	5.2	8.2	0.0289
Creatinine	1.3	1.2	1.5	1.7	1.4	2.0	1.4	1.3	1.5	0.0550
BUN	22.4	20.8	23.9	37.6	32.1	43.0	24.4	22.8	26.0	<0.0001
TIMP1	294.8	273.1	316.6	497.5	411.8	583.2	320.1	297.7	342.5	<0.0001
TIMP2	71.8	68.8	74.9	87.3	76.1	98.4	73.7	70.7	76.7	0.0009
MMP2	229.8	223.3	236.4	265.7	243.9	287.6	234.2	227.8	240.5	0.0003
MMP9	907.8	862.6	953.0	651.7	511.1	792.3	876.9	833.3	920.5	0.0002
MMP9/TIMP1	6.3	4.8	7.7	2.4	1.5	3.3	5.8	4.5	7.0	0.0481
MMP2/TIMP2	4.0	3.7	4.2	4.0	3.3	4.8	4.0	3.7	4.2	0.8146

* Data for gender is displayed as percentage.

Blood Urea Nitrogen (BUN), Matrix Metalloproteinase (MMP), tissue inhibitor of metalloproteinases (TIMP), C-reactive protein (CRP).

Of the 563 patients, the mean age was 57.6 years, ranging from 18 to 101 years. 52.0% of participants were men (95% CI 48.1–55.8). The mean age of men was 55.9 years (95% CI 53.8–57.9), the mean age of women was 59.5 years (95% CI 57.4–61.5), P = 0.0171. 68 patients (12.1%) died within the first 30 days of hospitalization in the ICU.

53.3% (95% CI 42.8–63.5) of non-survivors were men, while 51.7% (95% CI 47.6–55.8) of survivors were men, P = 0.7797. The mean age of non-survivors was 68.5 years (95% CI -71.9 65.1) and the mean age of survivors was 55.9 years (95% CI 54.3–57.5), P = <0.0001.The mean values for SOFA, APACHE and Charlson scores was lower in survivors than in non-survivors, P = <0.001 (1.7 (95% CI 1.5–1.9) vs 4.7 (95% CI 3.8–5.6), 9.4 (95% CI 8.9–9.9) vs 16.8 (95% CI 14.9–18.8) and 1.6 (95% CI 1.4–1.7) vs 2.7 (95% CI 2.1–3.3) respectively).

The mean values for C-reactive protein, procalcitonin, creatinine and blood urea nitrogen (BUN) were higher in survivors than non-survivors (130.3 (95% CI 121,4–139.2) vs 166.6 (95% CI 135.2–198.0) P = 0.0142, 6.1 (95% CI 4.6–7.6) vs 11.1 (95% CI 5.6–16.7) P = 0.0289, 1.3 (95% CI 1.2–1.5) vs 1.7 (95% CI 1.4–2.0) P = 0.0550 and 22.4 (95% CI 20, 8–23.9) vs 37.6 (95% 32,1–43.0) P = <0.0001 respectively).

The mean values for TIMP1, TIMP2 and MMP2 were lower in survivors than non-survivors (294.8 (95% CI 273.1–316.6) vs 497.5 (95% CI 411.8–583.2) P = <0.0001, 71.8 (95% CI 68.8–74.9) vs 87.3 (95% CI 76.1–98.4) P = 0, 0009 and 229.8 (95% CI 223.3–236. 4) vs 265.7 (95% CI 243.9–287.6) P = 0.0003, respectively), MMP9 was higher in survivors than in non-survivors (907.8 (95% CI 862,6–953.0) vs 651.7 (95% CI 511.1–792.3) P = 0.0002 respectively).

**Fig 1:**
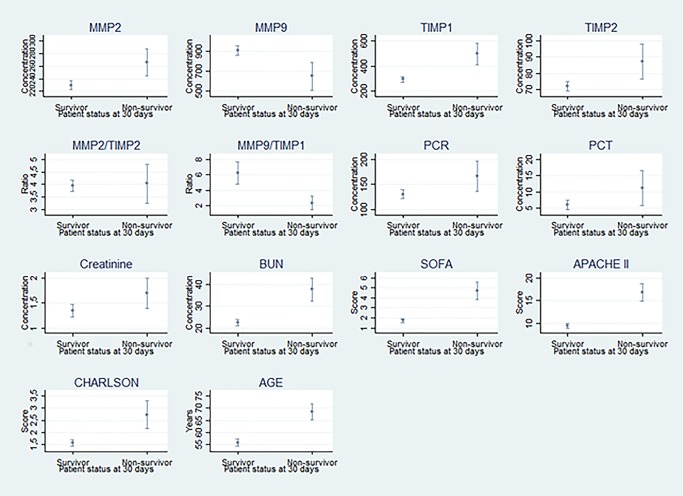
Mean laboratory and severity scale values stratified by survival.

Fig 1 shows that the mean values of C-reactive protein, procalcitonin, BUN, age, TIMP1, TIMP2, MMP2, SOFA scale, APACHE scale, and Charlson scale were higher in non-survivors compared with survivors at 30 days and the difference was statistically significant with an alpha <0.05.

The mean values of MMP9 and MMP9/TIMP1 ratio were higher in survivors vs non-survivors and the difference was statistically significant with an alpha of 0.05.

No statistically significant differences were found in gender, MMP2/TIMP2 ratio and serum creatinine when comparing survivors with non-survivors at 30 days.

The central point represents the mean and whiskers represents the 95% confidence interval.

Table 2 shows that the ability of each of the matrix metalloproteinases and their inhibitors to discriminate patient's death within the first 30 days is poor (Area under curve <70%).

**Table 2:** Discriminative capacity of the measured plasma biomarkers.

Variable	AREA UNDER CURVE (%)	CI 95% OF THE AREA UNDER THE CURVE
TIMP2	61.93	54.7–69.1
MMP9	33.7	26.1–41.5
TIMP1	68.8	61.9–75.6
TIMP2	59.5	52.0–66.9
MMP2/TIMP2 ratio	47.4	40.0–54.8
MMP9/TIMP1 ratio	25.2	18.9–31.5
CRP	59.9	52.0–67.7
PCT	69.9	64.6–75.2
CREATININE	62.0	55.3–68.7
BUN	74.8	69.8–79.7
SOFA score	74.5	69.0–80.1
APACHE II score	75.6	70.2–81.0
CHARLSON score	63.3	57.2–69.4

Matrix Metalloproteinase (MMP), tissue inhibitor of metalloproteinases (TIMP), C-reactive protein (CRP), Procalcitonin (PCT), Blood Urea Nitrogen (BUN).

The bivariate analysis was performed using logistic regression with a single independent variable against survival at 30 days. Results shown in Table 3 show that initially, with the exception of serum creatinine, gender and MMP2/TIMP2 ratio values, variables are statistically associated with mortality at 30 days.

**Table 3:** Bivariate analysis.

Variable	P	ODDS RATIO
APACHE II score	<0.0001	1.136
SOFA score	<0.0001	1.286
Age (years)	<0.0001	1.041
MMP9/TIMP1 ratio	<0.0001	0.775
BUN	<0.0001	1.027
TIMP1	<0.0001	1.002
CHARLSON score	<0.0001	1.270
MMP9	0.0001	0.999
MMP2	0.0005	1.005
TIMP2	0.0016	1.011
CRP	0.0174	1.003
Procalcitonin	0.047	1.012
Creatinine	0.0753	1.121
Gender	0.7796	0.938
MMP2/TIMP2 ratio	0.8161	1.011

Matrix Metalloproteinase(MMP), tissue inhibitor of metalloproteinases(TIMP), C-reactive protein(CRP), Blood Urea Nitrogen(BUN).

The different variables analyzed in the study are shown in Table 3 with their respective "P" and "OR" value. For APACHE score, SOFA score, CHARLSON score, age, MMP9/TIMP1 ratio, BUN, TIMP1 a P = <0.0001 with an OR of 1.136, 1.286, 1.270, 1.041, 0.775, 1,027 and 1,002 was obtained respectively. For MMP9 (P = 0.0001, OR = 0.999), MMP2 (P = 0.0005, OR = 1.005), TIMP2 (P = 0.0016, OR = 1.011), CRP (P = 0.0174, OR = 1.003), procalcitonin (P = 1.047, OR = 1.012), creatinine (P = 0.0753, OR = 1.121), gender (P = 0.7796, OR = 0.938), and MMP- 2/TIMP2 ratio (P = 0.8161, OR = 1.011).

**Fig 2:**
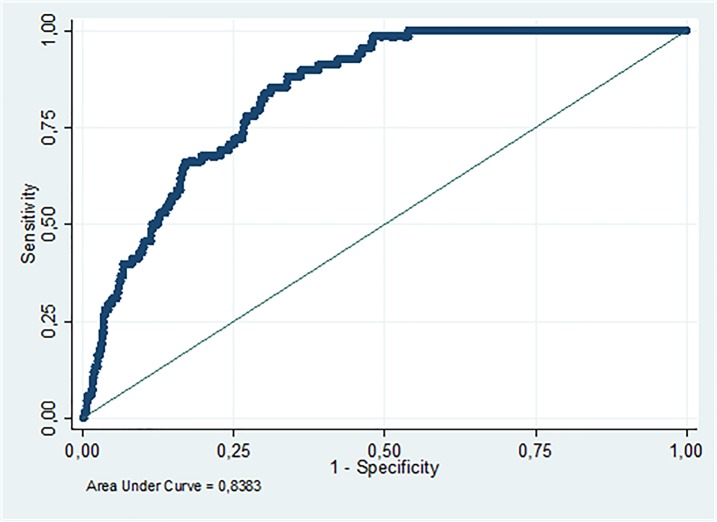
ROC curve from the multivariate model. Area under the curve of the multivariate model (SOFA score, age, TIMP1. CHARLSON score, MMP9).

A logistic regression model was built with a purposeful selection of covariates (Table 4) which showed that age, SOFA and Charlson scores, along with TIMP1 concentration, were statistically associated with mortality at 30 days of septic patients; serum MMP9 was not statistically associated with mortality of patients, but was a confounder of the TIMP1 variable which is why it was kept in the model. This model has a p = 0.2449 in the Hosmer-Lemeshow test which allows us to state that the data was adjusted appropriately to the model. In addition to assessing the discriminative ability of the model, we found that it had an area under the curve of 83.83% (Fig 2), which is considered an adequate discriminative ability.

**Table 4:** Final logistic regression.

VARIABLE	OR	P	CI 95%	
**SOFA score**	1.20	0.0000	1.11	1.30
**AGE (YEARS)**	1.03	0.0000	1.02	1.05
**TIMP1**	1.00104	0.0180	1.0002	1.0019
**CHARLSON score**	1.15	0.0430	1.00	1.32
**MMP9**	0.9994445	0.0500	0.9988887	1.000001

tissue inhibitor of metalloproteinases (TIMP), Matrix Metalloproteinase (MMP).

## Discussion

Currently mortality remains very high in sepsis and even more so in septic shock. In addition, diagnosis and course definition are complex due to the multiple factors involved [19]. Current studies suggest that people with a pre-existing chronic disease, people of older age, male gender and/or black race with an acute infection deteriorate faster and therefore are more susceptible to develop severe sepsis. Furthermore, the epidemiology of severe sepsis in developing countries may differ significantly from developed countries, which should be defined and taken into account in future research [20]. Hence, it represents a challenge to find biomarkers that improve diagnostic accuracy, define the course, timing and type of therapeutic intervention [4, 21].

MMPs and TIMPs are important in maintaining the balance of extracellular matrices in different tissues [5]. Specifically in sepsis, they have been involved in hepatic injury [22], acute lung injury [23], vascular [24] and cardiac dysfunction [25], multiple organ failure [26], impairment in the control of infection [27] and alterations in coagulation [28], which increase the odds of death [29]. Additionally, studies to date involving septic patients have shown an association between MMPs, TIMPs and mortality. However, the degree and direction of this association is not clear.

Our most prominent results include the finding of predictive values of MMP9 and TIMP1 plasma levels for mortality in septic patients enrolled in this study. It is worth mentioning that the number of patients enrolled in this study is higher than previous ones [13, 14, 15, 16, 30]. In a study of 20 patients with septic shock, Nakamura et al. [30], found levels of MMP9 significantly higher in non-survivors. Furthermore, in a study with 360 septic patients, Wang et al. [16], found significantly elevated levels of MMP9 in non-survivors. Hoffmann et al., and Lauhio et al., found no significant differences in the levels of MMP9 associated with mortality [13, 31].

These results differ from those of Lorente et al., 2009 [14], who observed (in the 192 patients studied with severe sepsis) significantly lower levels of MMP9 in non-survivors. In our study, like Lorente et al. [14], we found in a large group of septic patients, significantly lower levels of MMP9 in non survivors in the bivariate model. However, in the multivariate prediction model of mortality, it was determined as a confounding variable.

Like our study, other studies [14, 15, 16, 31] found significantly elevated levels of TIMP1 in non-survivors, which is worth mentioning (outstanding) since this finding in different studies is more consistent compared to MMP9. It is important to take into account that levels of TIMP1 in septic patients have been significant predictors of mortality in multivariate models, which suggests a crucial role of this biomarker in the pathophysiology and outcome of sepsis. This might suggest that the predictive value of MMP9 is probably related to their balance or counterbalance with TIMP1. However, it is important to mention that in our study MMP9/TIMP1 ratio had no predictive value in our multivariate prediction model of mortality.

Lorente et al. 2014, found a significant relationship between mortality and elevated levels of TIMP1/MMP9 ratio in the first, fourth and eighth day of the study. Furthermore, they presented three multivariate prediction models of mortality that include TIMP1/MMP9 ratio, diabetes and SOFA score measured at the first, fourth and eighth day [15].

Our study and Wang et al. 2014, which both involve a significant amount of septic patients (563 and 360, respectively), didn't corroborate the findings of Lorente et al. 2014, related to the predictive value of TIMP1/MMP9 ratio.

Here, we observe in the bivariate model, a significant relationship between MMP9/TIMP1 ratio and mortality, however, in the multivariate model, a significant relationship was not found. Likewise, Wang et al, 2014, didn't find a significant difference between MMP9/TIMP1 ratio values and the different degrees of sepsis severity, or between survivors and non-survivors [16].

Also, the triple model (which includes: sofa, diabetes and TIMP1/MMP9 ratio at the first, fourth, and eighth day [15], does not indicate the AUC of the predictive model, it only shows the AUC of TIMP1/MMP9 ratio, which are not as significant (<70%). Unlike our study, in which the AUC of MMP9/TIMP1 ratio is very low (25.2 with 95% CI = 18.9 to 31.5). Additionally, this triple model [15] would be impractical in the clinical setting. Our multivariate model has an AUC> 80%, which results from the measure taken at the time of the patient’s admission to the emergency department or intensive care unit.

Furthermore, Lorente et al., 2014 [15], found in the multivariate model diabetes as a significant comorbidity. In our study, not only did we take into account this comorbidity but we also took into accout the Charlson score (scale of comorbidities), which was significantly related with mortality in both the bivariate and multivariate model analyzes. Consequently the influence of chronic diseases in septic patients was better analyzed. In addition, the AUC value for TIMP1/MMP9 ratio found was below 70% [15], therefore it was not as significant. Additionally, MMPs and TIMPs were measured in serum and not plasma, but it is preferred in plasma because there could be a release of several MMPs or TIMPs from platelets in serum [32].

It is important to note that in our study, a multivariate logistic regression model was constructed by the method of intentional selection of covariates, initially including variables with P <0.2 in the bivariate analysis, obtaining the end model with 4 statistically significant variables (SOFA score, age, Charlson score, TIMP1) and a confounding variable (MMP9). Consequently, the predictive significance of both TIMP1 and MMP9 were striking, considering they were even superior to the APACHE II score and procalcitonin (PCT). Similar to ours, the multivariate model by Lorente et al., [14], showed that APACHE II score had no significant predictive value, unlike SOFA score and TIMP1. However, unlike ours, this model presents an AUC less than 70% for each of the variables, therefore a low predictive capacity. The AUC of our multivariate model was higher than 80%. It is important to highlight that Hoffmann et al. and Wang et al., [13, 16] did not adjust the analysis to the severity scales used in critically ill patients such as SOFA score, APACHE II score; Wang et al., only used Mortality in Emergency Department Sepsis (MEDS) score [16]. Thus, defining the size and scope of the biomarkers is more complicated taking into account that the degree of sepsis severity has a high probability of affecting the biomarkers studied.

So what is the biological substrate of these findings? Part of the answer is described in the pathophysiological role of MMP9 in myocardial dysfunction [25], vascular hyporeactivity [33], acute lung injury [23] and multiple organ dysfunction syndrome [26], undoubtedly explaining its association with mortality. Nevertheless, our results suggest that TIMP1 seems to have a more important biological role than MMPs, considering its association in the multivariate prediction model of mortality. TIMP1 is a nonselective enzymatic regulator of MMPs 2 and 9 and other MMPs [6], it is likely that, by its regulatory role on various types of MMPs, have pathophysiological effects outweigh alterations MMPs. Additionally, TIMP1 regulates the function of other non-MMP enzymes, which have been recently implicated in coagulation disorders [28] and prothrombotic state [15]. Hence, changes in TIMP1 can have broad and deep biological effects, whether it is associated or not with MMPs. More importantly, our results suggest that TIMP1 could possibly be a good biomarker for prediction of mortality regardless of the severity of the patient at time of admission. Additional studies that include serial quantifications of these markers are necessary for a better understading of their biomarker roll.

## Conclusions

Taking everything into account, our study presents a multivariate model with five variables, four significant variables which include TIMP1, and MMP9 as a confounding variable with potential use in clinical practice. Therefore, and considering the size of the patient population and the multivariate prediction model of mortality used here, it could be argued that plasma levels of TIMP1 should be considered as a promising prognostic biomarker in the setting of sepsis. Additionally, this study like other studies with large numbers of septic patients does not support the predictive value of TIMP1/MMP9 ratio. Further studies are required to better define the pathophysiological role of TIMP1 and how it could be a therapeutic target.

## Supporting information

S1 File Data base in Format stata 14. (DTA)

S2 File Data base in Format stata 11. (DTA)

S3 File Data base in Excel. (XLS)

S4 File Data bae in SPSS. (SAV)
